# Vitamin D Deficiency and Its Association With Vitamin D Receptor Gene Variants Among Malaysian Women With Hypertensive Disorders in Pregnancy: Protocol for a Nutrigenomics Study

**DOI:** 10.2196/53722

**Published:** 2024-03-26

**Authors:** Yakubu Ibrahim, Nurul Iftida Basri, Norshariza Nordin, Amilia Afzan Mohd Jamil

**Affiliations:** 1 Department of Obstetrics and Gynaecology Faculty of Medicine and Health Sciences Serdang Malaysia; 2 Department of Medical Laboratory Science Faculty of Allied Health Sciences, College of Medical Sciences Ahmadu Bello University Zaria, Kaduna State Nigeria; 3 Department of Biomedical Sciences Faculty of Medicine and Health Sciences Universiti Putra Malaysia Serdang Malaysia

**Keywords:** gestational hypertension, preeclampsia, hypertensive disorder, vitamin D deficiency, vitamin D receptor gene polymorphism, vitamin D sequence

## Abstract

**Background:**

Vitamin D deficiency has been associated with hypertensive disorders in pregnancy (HDP). The risk of developing HDP was reported to be further augmented among individuals with a vitamin D receptor (VDR) genetic variant. However, the reported roles of *VDR* variants in hypertensive disorders are inconsistent among different populations. Given the relatively higher incidence of vitamin D deficiency among Malaysian pregnant women and the high incidence of HDP in this population, we hypothesize that there may be associations between the risk of vitamin D deficiency and HDP with VDR genetic variants.

**Objective:**

This paper outlines the protocol for a study to determine the association of vitamin D status and *VDR* sequence variants among Malaysian pregnant women with HDP.

**Methods:**

This prospective study consists of two phases. The first phase is a cross-sectional study that will entail gathering medical records, a questionnaire survey, and laboratory testing for vitamin D status, with a planned recruitment of 414 pregnant women. The questionnaire will be utilized to assess the risk factors for vitamin D deficiency. The vitamin D status will be obtained from measurement of the vitamin D (25-hydroxyvitamin D_3_) level in the blood. The second phase is a case-control study involving a Malay ethnic cohort with vitamin D deficiency. Participants will be divided into two groups with and without HDP (n=150 per group). Genomic DNA will be extracted from the peripheral blood monocytes of participants using the Qiagen DNA blood kit, and *VDR* sequence variants will be determined using polymerase chain reaction–high-resolution melting (PCR-HRM) analysis. Sanger sequencing will then be used to sequence randomly selected samples corresponding to each identified variant to validate our PCR-HRM results. The *VDR* genotype and mutation frequencies of *BsmI*, *ApaI, TaqI,* and *FokI* will be statistically analyzed to evaluate their relationships with developing HDP.

**Results:**

As of December 2023, 340 subjects have been recruited for the phase 1 study, 63% of whom were determined to have vitamin D deficiency. In the phase 2 study, 50 and 22 subjects have been recruited from the control and case groups, respectively. Recruitment is expected to be completed by March 2024 and all analyses should be completed by August 2024.

**Conclusions:**

The outcome of the study will identify the nonmodifiable genetic components contributing to developing vitamin D deficiency leading to HDP. This will in turn enable gaining a better understanding of the contribution of genetic variability to the development of HDP, thus providing more evidence for a need of customized vitamin D supplementation during pregnancy according to the individual variability in the response to vitamin D intake.

**Trial Registration:**

ClinicalTrials.gov NCT05659173; https://clinicaltrials.gov/study/NCT05659173

**International Registered Report Identifier (IRRID):**

DERR1-10.2196/53722

## Introduction

Vitamin D deficiency is a global health issue affecting an estimated 1 billion people worldwide across all ethnicities and age groups [1]. Thus, vitamin D deficiency has become a pandemic despite the availability of sunlight in Asia, Africa, the Middle East, and Latin America. The most vulnerable populations at risk of vitamin D deficiency are pregnant women and their fetuses. Several studies have linked low vitamin D status with a higher risk of adverse short- and long-term health outcomes. In addition, risk factors such as dietary habits, cultural and religious practices such as wearing dark veils covering nearly all body parts that discourage sun exposure, and lack of government regulations for vitamin D fortification of foods further worsen the condition [2,3]

Hypertensive disorders in pregnancy (HDP) account for approximately 14% of all causes of maternal mortality globally. The spectrum of HDP includes gestational hypertension (GH); preeclampsia; eclampsia; and hemolysis, elevated liver enzymes, and low platelets (HELLP) syndrome. Preeclampsia is a complication of 2%-8% of all pregnancies worldwide, contributing to 16%, 9%, and 1.6%-2.5% maternal deaths globally, in Asia and Africa, and in Malaysia, respectively [4,5]. HDP is one of several disorders associated with vitamin D deficiency [6]. Vitamin D exerts its effect through the nuclear vitamin D receptor (VDR), and several single-nucleotide variants (SNVs; formerly single-nucleotide polymorphisms or SNPs) occur on the VDR gene, such as *BsmI, ApaI, TaqI*, and *FokI* SNVs. The *BsmI* and *FokI* variants of *VDR* have been reported to affect vitamin D binding and are associated with an increased risk of HDP [7].

In pregnant women, maternal 25-hydroxyvitamin D (25(OH)D) can freely cross the human placenta that expresses VDR and the enzyme CYP27B1, which converts 25(OH)D to its biologically active form 1,25-dihydroxycholecalciferol [8]. VDR is a protein comprising two functional domains (the N-terminal dual zinc finger DNA-binding domain and the C-terminal ligand-binding activity domain) and a linking region [9,10]. The gene encoding VDR is located on chromosome 12 (12q12-14) [11,12]. Several SNVs in the VDR gene have been associated with metabolic disorders and vitamin D deficiency to date, including rs1544410 (*BsmI*), rs7975232 (*ApaI*), and rs731236 (*TaqI*) [12].

Sequence variants in the VDR gene are associated with the dysregulation of metabolic biomarkers such as anthropometric parameters related to insulin resistance, obesity, and cardiovascular diseases, along with atherogenic lipid abnormalities in different populations [12,13]. Such metabolic dysregulation ultimately translates into complications in pregnancy with adverse health consequences for both the pregnant woman and the fetus. Vitamin D exerts its effect through the nuclear VDR. The *TaqI* variant, located at the 3′ untranslated region of the VDR gene, has been shown to affect mRNA stability and VDR expression in tissues. An SNV in *FokI* located near the promoter region causes altered VDR activity due to the change in the amino acid sequence of the protein [14]. A *FokI* variant of the VDR gene is also associated with upregulation of angiotensin II type I receptor and renin gene transcription, leading to hypertension [15]. A variant *BsmI* allele also influences *VDR* mRNA stability, resulting in a reduction in the amount of VDR protein produced in the tissues [16].

Accumulating evidence suggests that genetic variability involving variants of specific maternal susceptibility genes, such as those in the VDR gene, play vital roles in the pathogenesis of HDP [6]. An unfavorable *VDR* genetic background can significantly decrease the effectiveness of vitamin D action, thereby contributing to the development of HDP [6,17].

Moreover, the presence of a *VDR* sequence variant constitutes an important factor contributing to the individual susceptibility to the biological effects of vitamin D. HDP may be apparent in some pregnant women, whereas others present with a relatively milder sequela. Considering the influence of genetic variability and the roles of *BsmI, ApaI, TaqI*, and *FokI* variants in the etiopathogenesis of HDP, we aim to explore the possible association between the frequencies of these genetic variants and the incidence of HDP in Malaysian pregnant women. Given the estimated high prevalence of vitamin D deficiency among Malaysian pregnant women, we hypothesized that there are sequence variants in the *BsmI* and *FokI* VDR gene fragments (as the most common genotypes associated with HDP) of Malaysian pregnant women with vitamin D deficiency. We further explored associations with *ApaI* and *TaqI* variants given the relative lack of previous research on the potential associations of these genotypes with HDP.

## Methods

### Objectives

The objectives of this prospective study are to (1) determine the prevalence of vitamin D deficiency and associated risk factors among Malaysian pregnant women; (2) determine the frequencies of *BsmI*, *ApaI, TaqI,* and *FokI* genotypes among Malay pregnant women with vitamin D deficiency and HDP; (3) understand and associate the distributions of *VDR* genotype and allele frequencies in relation to vitamin D deficiency among Malay pregnant women; and (4) identify mutations in the *BsmI*, *ApaI, TaqI,* and *FokI* VDR gene fragments among Malay pregnant women with HDP.

This study is divided into two phases. Phase 1 is a cross-sectional study involving Malaysian pregnant women to achieve objective 1, whereas phase 2 is a case-control study among Malay pregnant women to achieve objectives 2, 3, and 4.

### Settings

This study will be carried out at Hospital Sultan Abdul Aziz Shah (HSAAS), a tertiary university hospital in Selangor, Malaysia. The hospital provides a range of health care services to the neighboring community and serves as the teaching hospital for the Faculty of Medicine and Health Sciences, Universiti Putra Malaysia. All pregnant women attending the Obstetrics and Gynaecology Department of HSAAS who fulfill the inclusion and exclusion criteria will be recruited between November 1, 2022, and March 31, 2024. A systematic random sampling method will be used to recruit the study participants for both the cross-sectional and case-control studies.

### Cross-Sectional Study (Phase 1)

#### Participants and Data

To achieve objective 1, Malaysian pregnant women, at any stage of gestation, with a singleton viable pregnancy at the time of recruitment and who are literate in the English or Malay language will be recruited. Women with a multiple pregnancy and on vitamin D supplementation are excluded. The gestation stage will be determined from the first day of the last menstrual cycle or based on measurement of the fetal crown-rump length determined on an ultrasound scan. Pregnant women taking vitamin D supplements or those who have been diagnosed with chronic diseases that are known to affect vitamin D levels, such as autoimmune disease and cancer, are excluded from the study.

Clinical, sociodemographic, dietary intake, and anthropometric (height, weight, and BMI) data will be collected using a pro forma. The prepregnancy BMI (kg/m^2^) will be calculated by dividing the weight (kg) by the square of the height (m^2^). Weight will be determined from prepregnancy body weight recall or obtained from the woman’s antenatal booking record along with the measured height. Participants will then be interviewed by a single trained interviewer to answer a validated questionnaire to determine their risk factors associated with vitamin D deficiency.

#### Validation of the Questionnaire

The questionnaire was adapted from the studies of Syed Nor et al [18], Jamil et al [19], and Humayun et al [20] (see Multimedia Appendix 1). Permission was obtained from all authors to adopt their questionnaires and translate them into the Malay language. The translation will be performed by two English-Malay-English translators before the validation test. A pilot test will be carried out among 40 pregnant women (approximately 10% of the calculated sample size) to assess the questionnaire’s clarity of meaning, appropriateness of the words used, and its cultural acceptance. Participants recruited for the pilot testing will not be included in the study. The reliability will be measured by calculating the internal consistency and assessing the associations among questions. A Cronbach α value of at least 0.7 indicates good internal consistency of the questionnaire [21].

In addition, the content validity index (CVI) will be calculated to measure the validity of the questionnaire where the relevancy of questions will be assessed by three experts in the field. The item-level CVI will be calculated by assessing each question’s validity, in which a score above 0.78 is considered acceptable [22]. The overall validity of the questionnaire will also be measured by calculating the scale-level CVI, in which a score above 0.9 is considered good [22].

#### Sampling Process

Approximately 5 milliliters of blood will be withdrawn and dispensed into a plain blood bottle for the measurement of 25(OH)D_3_ using an electrochemiluminescence immunoassay (Elecsys Vitamin D Total III assay). This assay kit includes a vitamin D–binding protein (VDBP) labeled with a ruthenium complex as the capture protein to bind 25(OH)D_3_ and 25(OH)D_2_. Cross‑reactivity to 24,25(OH)D is blocked by a specific monoclonal antibody.

The sample will first be incubated with pretreatment reagents 1 and 2, where bound 25(OH)D is released from the VDBP. Subsequently, the pretreated sample is incubated with the ruthenium-labeled VDBP to form a complex between 25(OH)D and the ruthenylated VDBP. A specific unlabeled antibody binds to the 24,25(OH)D present in the sample and inhibits cross-reactivity to this vitamin D metabolite. During the third incubation, streptavidin-coated microparticles and 25(OH)D labeled with biotin are added. Unbound ruthenylated-labeled VDBPs become occupied, and a complex consisting of the ruthenylated VDBP and the biotinylated 25(OH)D is formed, which finally binds to the solid phase via the interaction of biotin and streptavidin.

The reaction mixture is aspirated into the measuring cell where the microparticles are magnetically captured onto the surface of the electrode. Unbound substances are then removed with ProCell/ProCell M. Application of a specific voltage to the electrode then induces chemiluminescent emission, which is measured by a photomultiplier. The results are determined via a calibration curve that is specifically generated by a 2‑point calibration process and compared to a master curve provided via the reagent barcode or e‑barcode.

#### Quality Control

Per-run control sera (deficient, insufficient, and normal) will be used for sample analysis. All laboratory analytical protocols will be performed with strict adherence to the quality control measures.

#### Classification of Vitamin D Status

The results will be further classified according to vitamin D status as deficient (˂30 nmol/L), insufficient (30-50 nmol/L), and normal (≥50 nmol/L), using the 2011 classification guidelines of the Institute of Medicine [23].

### Case-Control Study (Phase 2)

#### Participants and Data

For the second phase of the study, we chose to focus on the Malay ethnic group, who make up the majority of the Malaysian population. Pregnant women of Malay ethnicity who are classified in the vitamin D deficiency group in phase 1 of the study will be recruited for phase 2. Those with a nonviable pregnancy or those who received a diagnosis of chronic hypertension prior to the current pregnancy will be excluded. The remaining participants will then be divided into two groups: those with HDP (GH, preeclampsia, eclampsia, HELLP syndrome) and those with blood pressure in the normal range.

#### Blood Collection and DNA Extraction

Approximately 5 milliliters of blood will be collected into ethylenediaminetetraacetic acid (EDTA) containers for genetic analysis. The blood samples will be transported to the laboratory under a cold chain. Immediately after collection, the genomic DNA will be extracted using the QIAamp DNA blood kit (Qiagen). The extracted DNA will be checked for purity using an ultraviolet spectrophotometer and the integrity will be checked by running the genomic DNA on a 2% agarose gel using triacetate-EDTA buffer in parallel with DNA ladders (all procured from Vivantis Sdn Bhd Malaysia). The genomic DNA with high quality will be stored at –20 °C pending genetic analysis.

#### VDR Genotyping

The polymerase chain reaction–high-resolution melting (PCR-HRM) technique will be used to amplify the VDR gene with specific primers. The amplified region of the VDR gene (amplicon) will be stored in a PCR tube. Sets of primers (procured from Apical Scientific and Biotechnology Sdn Bhd Malaysia) spanning the 5′-3′ region of the *VDR* sequence will be synthesized based on published sequences [24], which will be used to run the PCR-HRM analysis to detect *VDR* variants. The extracted DNA will be subjected to PCR-HRM following the manufacturer’s protocol using the cycling conditions based on a previous report [24].

#### Principle of PCR-HRM

PCR-HRM analysis is a sensitive and specific technique for the detection of mutations on double-stranded DNA (dsDNA) samples (amplicons). First, the region of interest on the DNA is amplified in real time using specific primers prior to the HRM phase. The HRM process then begins with slow denaturation of the dsDNA with a temperature range of 50-95 °C in conjunction with an intercalating fluorescent dye, SYBR Green (Roche). When the melting temperature of the dsDNA is reached, the two strands “melt” apart. The midpoint of the melt curve is described as the point when 50% of the DNA is double-stranded and 50% is single-stranded. The shape of the curve is dependent on the characteristics of the dsDNA, which relate to whether it is the homozygous wild-type, homozygous mutant, or heterozygous wild-type and mutant genotype. When the two strands “melt” apart, the fluorescence level drops. As the HRM is monitored in real time, this curve offers a real-time picture of the characteristics of the DNA being tested.

#### Barcode-Tagged Sequencing Analysis

Barcode-tagged sequencing (BT-Seq) analysis, as a next-generation sequencing approach, will be used to verify the *VDR* SNVs identified among the Malay pregnant women with vitamin D deficiency and HDP according to the PCR-HRM results. BT-Seq services will be sought from TreeCode Sdn Bhd Malaysia. Ten amplicon samples will be selected from each of the identified *VDR* variants as representative samples to serve as the reference genotype for all SNVs detected in the PCR-HRM analysis. The analysis will be performed on each *VDR* variant amplicon that will be synthesized based on published primers spanning the four known VDR SNVs (*BsmI, ApaI, TaqI,* and *FokI*) using PCR [24]. The amplicons will be run on an agarose gel and the purified products will be cut from the gel before sending to the company providing the BT-Seq service. Discrimination of the three possible genotypes of each genetic variant (common homozygotes, heterozygotes, and rare homozygotes) in 4 distinct groups will be obtained from 180 samples by PCR-HRM analysis. A T-to-C transition in introns 8 and 9 will reflect the presence of SNVs in introns 8 and 9 for *BsmI* and *TaqI*, respectively; a C-to-T transition at the junction of intron 1 and exon 2 reflects the *FokI* variant; and the *ApaI* variant is reflected by a T-to-G transition in intron 8. The corresponding distribution of vitamin D status and the genotype frequency in Hardy-Weinberg equilibrium will be obtained for each SNV.

### Sample Size Estimation

#### Total Sample Size

A total of 414 pregnant women will be recruited for the cross-sectional study (phase 1) to determine the prevalence of vitamin D deficiency and associated risk factors (objective 1). For phase 2, a total of 150 Malaysian pregnant women with HDP will be recruited as the case group and 150 normotensive Malaysian pregnant women with vitamin D deficiency will be recruited as controls.

#### Sample Size Calculation for the Cross-Sectional Study (Phase 1)

The sample size for the study was calculated using the following formula [25]:


n = (Z^2^ × p × q)/d^2^
**(1)**

where n is the minimum sample size, Z is the level of significance at the 95% CI (1.96), p is the prevalence rate, q = 1 − p, and d is the tolerable margin of error (5%).

According to Woon et al [26], the prevalence of vitamin D deficiency among Malaysian pregnant women is 42.6%. Applying equation 1, the sample size is calculated as follows:


n = (1.96)^2^ × 0.426 × (1 – 0.426)/(0.05)^2^

n **=** (3.8416 × 0.426 × 0.574)/0.0025 = 376

Therefore, the minimum sample size required for the cross-sectional study after addition of a 10% attrition rate is approximately 414 Malaysian pregnant women.

#### Sample Size Determination for the Case-Control Study (Phase 2)

The minimum number of participants to be recruited in the case-control study was determined using the following formula [27]:


n = r + 1 × P* × (1 – P*) × (Z_β_ + Z_α/2_)^2^/r × (P_1_ – P_2_)^2^
**(2)**

where r is the ratio of cases to controls (1 in this instance given the equal numbers in the two groups), P_1_ is the proportion of exposed individuals in the cases, P_2_ is the proportion of exposed individuals in the controls, P* is the effect size (P_1_ – P_2_), Z_β_ at 80% power is 0.84, and Z_α/2_ is 1.96 for a .05 significance level at the 95% CI.

According to Caccamo et al [6], the prevalence of vitamin D deficiency among women with GH (P_1_) and that among pregnant women without hypertension (P_2_) is 21% and 11%, respectively. Therefore, P* is determined to be 0.1 (0.21–0.11). Applying equation 2, the sample size is calculated as:


n = (1 + 1) × (0.1) × (1 – 0.1) × (0.84 + 1.96)^2^/1 × (0.1)^2^

n = 141

Considering dropout or missing samples, 10% attrition is added to the calculated sample size. Hence, our minimum number of participants in each group (cases and controls) was determined to be 150.

### Ethical Considerations

The research protocol has been approved by the Ethics Committee for Research Involving Human Subjects of the Universiti Putra Malaysia (reference number JKEUPM-2021-915). The study will be conducted in accordance with the standards of human experimentation in the Declaration of Helsinki and Malaysian Good Clinical Practice Guideline. This study protocol was also registered with ClinicalTrials.gov (NCT05659173). All eligible participants will be given a patient information sheet consisting of information about the study. Those who agree will be asked to sign a written consent form (available in both the Malay and English languages). Participation is voluntary and participants have the right to withdraw at any time without giving a reason. Any amendment to the protocol or documents will be submitted for review to the ethics committee. To preserve the privacy of the participants, any personal details will be removed from publications. Only anonymized and deidentified information will be made available in future manuscripts related to this work. All data of the study will be kept in a password-protected database on a password-protected desktop computer at the host organization and will only be accessible by the named researchers. As this is an observational cross-sectional and case-control study, there will be no compensation given to the participants given that the study is determined to be of low risk.

### Data Analysis Plan

Data obtained from the study will be analyzed using SPSS version 27.0 (IBM Corp). Data entered will be checked for missing or suspicious values. These will subsequently be verified with the participants or omitted as missing values. Descriptive analysis will be used to summarize the sociodemographic data, anthropometric data, clinical data, vitamin D dietary intake, sun exposure, and physical activity. The prevalence of vitamin D deficiency will be expressed in frequency and percentage. The association with sociodemographic data will be analyzed using the *χ*^2^ test, independent *t* test, and ANOVA where applicable.

We will report the comparative prevalence of *BsmI*, *ApaI, TaqI,* and *FokI* *VDR* genotypes among Malay pregnant women with vitamin D deficiency with and without HDP. Similarly, the distributions of *VDR* genotype and allele frequencies according to vitamin D deficiency among Malay pregnant women will be reported as a descriptive analysis. Any mutations found in the *BsmI*, *ApaI, TaqI,* and *FokI* VDR gene fragments among Malay pregnant women with HDP will also be described. The associations of vitamin D status, associated risk factors, and VDR genetic variants with HDP will be determined using multivariate logistic regression analysis. The level of significance will be considered at a threshold of *P*≤.05 at the 95% CI.

## Results

As of December 2023, a total of 340 subjects have been recruited for the phase 1 study. Preliminary analysis showed that 63% of the participants were vitamin D–deficient. Other analyses are pending. For the phase 2 study, 50 control subjects and 22 case subjects have been recruited to date. We are expecting to complete the recruitment by March 2024 and to complete all laboratory and questionnaire analyses by August 2024.

## Discussion

### Projected Significance

The study will provide information on the current prevalence and modifiable risk factors of vitamin D deficiency. The prospective associations of vitamin D deficiency with the study variables will be of immense benefit to physicians and public health experts in formulating policies related to vitamin D supplementation. The outcome will greatly advance our current understanding of vitamin D deficiency in Malaysia and at mechanistic level could be adopted by other low- and middle-income countries to tackle the widespread problem of vitamin D deficiency.

Additionally, this study holds promise for determining the influence of unchangeable genetic components that may impact vitamin D deficiency and its correlation with HDP. This will constitute a significant new development in the field. Determining the association of the genetic variants will enable gaining a further understanding of the roles of the nonmodifiable components contributing to the risk of vitamin D deficiency that lead to the development of HDP.

### Strengths and Limitations

The sample size of this study is one of the highest among similar studies conducted in the study area performed to date [7,17]. In addition, the assessment of both modifiable and nonmodifiable risk factors of vitamin D deficiency will facilitate gaining a deeper understanding of cause and effect, as these factors relate not only to vitamin D deficiency itself but also involve associated genetic variants and the risk of developing HDP.

Potential limitations include bias in recall in answering the vitamin D food frequency components of the questionnaire given that these data are based on the self-reporting of participants. However, efforts have been made to ensure that these challenges are significantly tackled by conducting pre-enrollment interviews with pregnant women for eligibility before their inclusion in the study. As the study will be conducted in a tertiary hospital setting and a convenience sampling strategy is used, only subjects of interest that suit our inclusion criteria will be recruited. Thus, the results of this study may be influenced by the recruited participants group, which may not necessarily replicate the entire Malaysian population. Future studies should adopt a systematic random sampling strategy with a larger sample size and multicentered design to capture pregnant women from broader demographics, including those living in rural areas.

### Conclusion

The outcome of this prospective study will determine both modifiable and nonmodifiable factors for vitamin D deficiency and will provide evidence to support targeted vitamin D supplementation programs. Furthermore, the study will help to enhance our understanding of the association of genetic variability in the VDR gene with the risk of developing HDP. These findings could bring together relevant stakeholders in government health authorities, food and drug administrations, and food manufacturing industries to contribute to the establishment of national intervention schemes for the screening, prevention, and treatment of vitamin D deficiency that would be customized for susceptible women of reproductive age.

## Appendix

Multimedia Appendix 1 Questionnaire.

Multimedia Appendix 2 Peer-reviewer report from the Ministry of Higher Education.

## Abbreviations

25(OH)D: 25-hydroxyvitamin D
BT-Seq: barcode-tagged sequencing
CVI: content validity index
dsDNA: double-stranded DNA
EDTA: ethylenediaminetetraacetic acid
GH: gestational hypertension
HDP: hypertensive disorders of pregnancy
HELLP: hemolysis, elevated liver enzymes, and low platelets
HSAAS: Hospital Sultan Abdul Aziz Shah
PCR-HRM: polymerase chain reaction–high-resolution melting
SNV: single-nucleotide variant
VDBP: vitamin D–binding protein
VDR: vitamin D receptor

## Data Availability

The data obtained and analyzed during the study will not be made publicly available due to privacy issues but will be available on reasonable request to the corresponding author.
