# Correction: Vitamin D Deficiency and Its Association With Vitamin D Receptor Gene Variants Among Malaysian Women With Hypertensive Disorders in Pregnancy: Protocol for a Nutrigenomics Study

**DOI:** 10.2196/59300

**Published:** 2024-04-11

**Authors:** Yakubu Ibrahim, Nurul Iftida Basri, Norshariza Nordin, Amilia Afzan Mohd Jamil

**Affiliations:** 1 Department of Obstetrics and Gynaecology Faculty of Medicine and Health Sciences Serdang Malaysia; 2 Department of Medical Laboratory Science Faculty of Allied Health Sciences, College of Medical Sciences Ahmadu Bello University Zaria, Kaduna State Nigeria; 3 Department of Biomedical Sciences Faculty of Medicine and Health Sciences Universiti Putra Malaysia Serdang Malaysia

In “Vitamin D Deficiency and Its Association With Vitamin D Receptor Gene Variants Among Malaysian Women With Hypertensive Disorders in Pregnancy: Protocol for a Nutrigenomics Study (JMIR Res Protoc 2024;13:e53722) the authors noted one error.

The author list was originally:

Yakubu Ibrahim^1,2^; Nurul Iftida^1^; Norshariza Nordin^3^; Amilia Afzan Mohd Jamil^1^

and has been changed to:

Yakubu Ibrahim^1,2^; Nurul Iftida Basri^1^; Norshariza Nordin^3^; Amilia Afzan Mohd Jamil^1^

The correction will appear in the online version of the paper on the JMIR Publications website on April 11, 2024, together with the publication of this correction notice. Because this was made after submission to full-text repositories, the corrected article has also been resubmitted to those repositories.

