# Supplementary material for: Vitamin D Deficiency and Its Association With Vitamin D Receptor Gene Variants Among Malaysian Women With Hypertensive Disorders in Pregnancy: Protocol for a Nutrigenomics Study
Source: JMIR Res Protoc. 2024 Mar 26;13:e53722. doi: 10.2196/53722 (PMC11005429; doi:10.2196/53722)
Supplement: Multimedia Appendix 1 [file resprot_v13i1e53722_app1.docx]

**Multimedia Appendix 1: QUESTIONNAIRE**

| Participant Code: ……………… | Date: ………………………… | |
| --- | --- | --- |
| **BIO-DATA** | | |
| Name: …………………………  Mobile Phone No.: …………………… | MRN: ………….......... | Date of Birth (Age): …………  I/C No.: …………… |
| **SOCIO-DEMOGRAPHIC DATA** | | |
| Educational status  Household Income Per month  Work status/Occupation  Marital status | Primary [ ] Secondary [ ] Diploma [ ] Tertiary [ ]  [ ] B40 (<RM4850)  [ ] M40 (between RM4851 to RM10970)  [ ] T20 (>RM10971)  [ ] Working  [ ] Unemployed  [ ] Retired  [ ] Student  [ ] House wife  [ ] Married  [ ] Single  [ ] Divorced  [ ] Widow | |
| **ANTHROPOMETRIC MESUREMENTS** | | |
| Height (cm) …………… | Booking weight (Kg) ………  Current Pregnancy weight (Kg) …………..  Booking BMI (Kg/m²) …………. | |
| **CLINICAL MEASUREMENTS/DATA** | | |
| Blood Pressure at recruitment  Last menstrual period (LMP)  Estimated date of delivery (EDD)  Gestation Age (weeks)  Gravidity and Parity  Family History of chronic diseases  Past pregnancy complications  Current pregnancy problem | Systolic BP (mmHg) …………..  Diastolic BP (mmHg) …………..  Date of LMP [………..]  Date of EDD [………..]  [………….]  […………]  [ ] Hypertension  [ ] Diabetes mellitus  [ ] Liver Disease  [ ] Kidney  [ ] Disease  [ ] Cancer  [ ] Heart Disease  [ ] Autoimmune Disease  [ ] Obesity  Others……………..  [ ] Gestational hypertension  [ ] Preeclampsia  [ ] Eclampsia  [ ] Gestational diabetes  Others………… | |
| **PREGNANCY PHYSICAL ACTIVITY QUESTIONS** | | |
| Preparing meals (cook, setting table, washing dishes)  Carrying children.  Sitting and reading, talking, or on the phone, while not at work.  Shopping (for food,clothes, or other items)  Light cleaning (make beds, laundry, iron)  Heavier cleaning (vacuum,mop,sweep,wash windows)  Mowing lawn while on a riding mower  Watching TV or a video  Walking slowly for fun or exercise  Walking more quickly for fun or exercise  Walking quickly up hills for fun or exercise  Jogging | [ ] None  [ ] Less than 30 minutes  [ ] 30 minutes – 1 hour  [ ] 1 hour – 2 hours  [ ] 2 hours – 3 hours  [ ] None  [ ] Less than 30 minutes  [ ] 30 minutes – 1 hour  [ ] 1 hour – 2 hours  [ ] 2 hours – 3 hours  [ ] None  [ ] Less than 30 minutes  [ ] 30 minutes – 1 hour  [ ] 1 hour – 2 hours  [ ] 2 hours – 3 hours  [ ] None  [ ] Less than 30 minutes  [ ] 30 minutes – 1 hour  [ ] 1 hour – 2 hours  [ ] 2 hours – 3 hours  [ ] None  [ ] Less than 30 minutes  [ ] 30 minutes – 1 hour  [ ] 1 hour – 2 hours  [ ] 2 hours – 3 hours  [ ] None  [ ] Less than 30 minutes  [ ] 30 minutes – 1 hour  [ ] 1 hour – 2 hours  [ ] 2 hours – 3 hours  [ ] None  [ ] Less than 30 minutes  [ ] 30 minutes – 1 hour  [ ] 1 hour – 2 hours  [ ] 2 hours – 3 hours  [ ] None  [ ] Less than 30 minutes  [ ] 30 minutes – 1 hour  [ ] 1 hour – 2 hours  [ ] 2 hours – 3 hours  [ ] None  [ ] Less than 30 minutes  [ ] 30 minutes – 1 hour  [ ] 1 hour – 2 hours  [ ] 2 hours – 3 hours  [ ] None  [ ] Less than 30 minutes  [ ] 30 minutes – 1 hour  [ ] 1 hour – 2 hours  [ ] 2 hours – 3 hours  [ ] None  [ ] Less than 30 minutes  [ ] 30 minutes – 1 hour  [ ] 1 hour – 2 hours  [ ] 2 hours – 3 hours  [ ] None  [ ] Less than 30 minutes  [ ] 30 minutes – 1 hour  [ ] 1 hour – 2 hours  [ ] 2 hours – 3 hours | |
| Dancing | [ ] None  [ ] Less than 30 minutes  [ ] 30 minutes – 1 hour  [ ] 1 hour – 2 hours  [ ] 2 hours – 3 hours | |
| Prenatal exercise class | [ ] None  [ ] Less than 30 minutes  [ ] 30 minutes – 1 hour  [ ] 1 hour – 2 hours  [ ] 2 hours – 3 hours | |
| Swimming | [ ] None  [ ] Less than 30 minutes  [ ] 30 minutes – 1 hour  [ ] 1 hour – 2 hours  [ ] 2 hours – 3 hours | |
| Sitting at working or in class | [ ] None  [ ] Less than 30 minutes  [ ] 30 minutes – 1 hour  [ ] 1 hour – 2 hours  [ ] 2 hours – 3 hours | |
| Walking quickly at work while carrying things (heavier than 4 litres jug of milk) | [ ] None  [ ] Less than 30 minutes  [ ] 30 minutes – 1 hour  [ ] 1 hour – 2 hours  [ ] 2 hours – 3 hours | |
| Standing or slowly walking at work while carrying things (heavier than 4 litres jug of milk) | [ ] None  [ ] Less than 30 minutes  [ ] 30 minutes – 1 hour  [ ] 1 hour – 2 hours  [ ] 2 hours – 3 hours | |
| Walking quickly at work not carrying anything | [ ] None  [ ] Less than 30 minutes  [ ] 30 minutes – 1 hour  [ ] 1 hour – 2 hours  [ ] 2 hours – 3 hours | |
| Standing or slowly walking at work not carrying anything | [ ] None  [ ] Less than 30 minutes  [ ] 30 minutes – 1 hour  [ ] 1 hour – 2 hours  [ ] 2 hours – 3 hours | |
|  | | |
|  |  | |
|  |  | |
|  |  | |
| **SUN EXPOSURE** | | |
| Type of dressing / body cover | [ ] None  [ ] Half body cover  [ ] Partial body cover  [ ] Full body cover | |
| **VITAMIN D DIETARY INTAKE/FOOD FREQUENCY** |  | |
| Fish (Salmon, Tuna, mackerel or sardine, etc) | [ ] 0 - 50g per serving  [ ] 50 - 100g per serving  [ ] 100 and above per serving  [ ] None  Frequency of intake (number of times per day: ………………. | |
| Dairy products (milk, butter and cheese or yoghurt) | [ ] 0 - 50g per serving  [ ] 50 - 100g per serving  [ ] 100 and above per serving  [ ] None  Frequency of intake (number of times per day: ………………. | |
| Vegetables (Mushrooms, oyster, Potatoes, mashed etc) | [ ] 0 - 50g per serving  [ ] 50 - 100g per serving  [ ] 100 and above per serving  [ ] None  Frequency of intake (number of times per day: ………………. | |
