# Supplementary material for: Vitamin D Deficiency and Its Association With Vitamin D Receptor Gene Variants Among Malaysian Women With Hypertensive Disorders in Pregnancy: Protocol for a Nutrigenomics Study
Source: JMIR Res Protoc. 2024 Mar 26;13:e53722. doi: 10.2196/53722 (PMC11005429; doi:10.2196/53722)
Supplement: Multimedia Appendix 2 [file resprot_v13i1e53722_app2.pdf]

MINISTRY OF HIGHER EDUCATION

SINGLE DISCIPLINARY PROJECT

APPLICATION FORM
FUNDAMENTAL RESEARCH GRANT SCHEME (FRGS)
Skim Geran Penyelidikan Fundamental
(Pindaan 1/2012)

JABATAN PENDIDIKAN TINGGI
KEMENTERIAN PENGAJIAN TINGGI

A. Application Details

Application ID	438015-462109
Reference Code	FRGS/1/2022/SKK01/UPM/02/1
A(i). Selected Grant Scheme	FRGS 2022-1
A(ii). Title of Proposed Research Project	Association of Vitamin D deficiency With Selected Vitamin D Receptor (VDR) Gene Polymorphisms In Gestational Hypertension Among Malaysian Women: A Prospective Genetic Biomarker For Early Intervention Strategy<br><br>*Previous title:*<br>*Association of Vitamin D deficiency With Selected Vitamin D Receptor (VDR) Gene Polymorphisms In Gestational Hypertension Among Malays: A Prospective Genetic Biomarker For Early Intervention Strategy*
A(iii). Keywords	vitamin D deficiency, genetic polymorphisms, vitamin D receptor(VDR) gene, gestational hypertension

B. Details of Project Leader

B(i). Name	Nurul Iftida Binti Basri
B(ii). Academic Qualification	Doctor of Obstetrics & Gynaecology UKM<br>MRCOG DRCOG London<br>MBCHB Dundee
B(iii). Nationality	Malaysia
B(iv). IC/Passport No.	840313025172
B(v). Position	Senior Lecturer
B(vi). Institution	Universiti Putra Malaysia (UPM)
B(vii). Faculty/Centre	Faculty of Medicine and Health Sciences, Obstetrics and Gynaecology
B(viii). Unit/Department	Obstetrics and Gynaecology
B(ix). Office Phone No.	0397699339
B(x). Handphone No.	0133452294
B(xi). E-mail Address	nurul.iftida@upm.edu.my
B(xii). Date of First Appointment with this Institution	01/08/2019
B(xiii). Type of Service (Permanent/Contract)	Permanent 13/03/2050

C. Research Information

C(i). Research Domain

Research Domain	Sub Research Domain
Clinical and Health Sciences	Clinical Studies

C(ii). Research Cluster

Cluster: Health

C(iii). 10-10 Malaysia Science, Technology, Innovation and Economy (MySTIE) - based on 10 Socio-Economic Drivers

MySTIE: Medical and Healthcare

C(iv). Shared Prosperity Vision 2030 (SPV 2030)

SPV: KEGA 14 – Advanced & Modern Services

C(v). Sustainable Development Goals (SDGs)

SDG: SDG 3 – Good Health and Well-being

C(vi). Location of Research

Location
Faculty of Medicine & Health Sciences (FMHS) UPM- Medical Genetics Lab
Agro Biotechnology Institute (ABI) Serdang, Selangor (liquid-chromatography mass spectrometry analysis machine)
Hospital Pengajar Universiti Putra Malaysia- Obstetrics and Gynaecology Department
Hospital Serdang- Obstetrics and Gynaecology Department

C(vii). Duration of this research

From	1/September/2022
To	31/August/2025
Duration	3 years

C(viii). Other Researchers *To add a project member from a government agency/foreign institution/industry, the project leader needs to register the new member if he/she is still not registered in MyGRANTS database. Please click the icon to search for existing or add new members.*

Researcher Id	Name	IC / Passport Number	Faculty/ School/ Centre/ Unit/ Department	Position	Area of Expertise	Next Appointed Leader	Role
18070	Norshariza Binti Nordin	710507075180	Universiti Putra Malaysia	Associate Professor (Dr)	Molecular Biology, Neural development, Stem cell, Stem Cells, Neuroscience, Molecular Medicine	☐	Guiding laboratory analysis during genomic DNA extraction, identification of polymorphic VDR gene variants and validation of SNPs by NGS
18932	Loh Su Peng	741129125006	Universiti Putra Malaysia	Associate Professor (Lecturer)	Nutrition	☐	Guiding the development of validated questionnaire and provide advice on publication work
19101	Amilia Afzan binti Mohd Jamil	770425145186	Universiti Putra Malaysia	Senior Lecturer (dr)	Obstetric and Gynaecology, obstetric and Gynaecology, perinatal medicine, women's pharmacology	☑	Main supervisor of PHD student, provide advice on technical and clinical issues
127232	Aida Adha Binti Mohd Jamil	811020145338	Universiti Tunku Abdul Rahman	Lecturer (Lecturer)	Data Science	☐	Providing assistance in questionnaire validation and medical statistics for data analysis and interpretation

C(ix). Research projects that have been completed or are on-going by project leader in the last three years

Title	Grant Name	Role	Progress (%)	Status	Duration	Start Date	End Date
Towards Development of An Individualised Screening Tool by Utilising Cervical Length Measurement in Relation to Body Mass Index For Early Identification and Intervention of Preterm Birth	Geran Putra IPM	Project Leader	N/A	In Progress	2 years	15/02/2021	14/02/2023
The Malaysian GestatiOnal Diabetes and prevention of DiabetES Study (MY GODDESS)	MYPAIR / KPM	Member	N/A	In Progress	3 years	01/01/2020	31/12/2022

C(x). Academic publications that have been published by the project leader in the last five years

Title	Name of Journal	Year
Prophylactic gonadectomy in 46 XY females; why, where and when?	Hormone Molecular Biology and Clinical Investigation	2021
Protocol for a Qualitative Study Exploring The Perception of Need, Importance and Acceptability of a Digital Diabetes Prevention Intervention For Women With Gestational Diabetes Mellitus During and After Pregnancy in Malaysia (Explore-MYGODDESS)	BMJ Open	2021
A Protocol of Process Evaluations of Interventions for the Prevention of Type 2 Diabetes in Women With Gestational Diabetes Mellitus: A Systematic Review	International Journal of Qualitative Methods	2021
Celecoxib Versus Mefenamic Acid in The Treatment of Primary Dysmenorrhea.	Hormone Molecular Biology and Clinical Investigation	2020
Unilateral Pleural Effusion: A Rare Presentation in Pregnancy	Journal of Obstetrics and Gynaecology	2020
Management Options and Outcomes of Cerebral Arteriovenous Malformation in Pregnancy: Case Series	Arch Iran Med	2019
Case Report: Congenital Complete Heart Block in Pregnancy. Nurul I Basri, Shuhaila Ahmad. Hormone Molecular Biology and Clinical Investigation	Hormone Molecular Biology and Clinical Investigation	2018
The World Health Organization (WHO) versus The International Association of Diabetes in Pregnancy Study Group (IADPSG) diagnostic criteria of gestational diabetes mellitus (GDM) and their associated maternal and neonatal outcomes	Hormone Molecular Biology and Clinical Investigation	2018

C(xi). Executive Summary of Research Proposal

(Please include the problem statement, objectives, research methodology, expected output/outcomes/implication, and significance of output from the research project)

Hypertensive disorders of pregnancy account for approximately 14% of maternal mortality globally and is the fourth leading cause of maternal mortality in Malaysia. Despite this, the mechanisms and pathogenesis are still unknown. Vitamin D deficiency (hypovitaminosis vitamin D) has been shown to be one of the causes of gestational hypertension(GH).

Several populations have observed the association of single nucleotide polymorphisms (SNPs) of vitamin D receptor (VDR) to vitamin D deficiency among pregnancy complications, including GH. Alarming cases of hypovitaminosis D in sunny areas including Malaysia, highly imply the need to understand the genetic factor. Hence, genotyping VDR variants among pregnant women is essential for early vitamin D supplementation strategy. To our knowledge, there has been no published study conducted among Malaysian population on the association of VDR genetic variation and GH. Therefore, we aim to investigate the prevalence of vitamin D deficiency and its association of VDR SNPs to the development of GH among Malaysian pregnant mothers, with the main focus on Malays, representing the largest ethnic in Malaysia.

The prevalence will be determined through a cross-sectional study involving 363 pregnant women recruited in HPUPM and Hospital Serdang, Selangor. Relevant socio-demographic, clinical and anthropometric data will be collected using structured interviewer-administered questionnaire. Blood specimens for the analysis of vitamin D will be done. The association will be determined in a case-control study involving 180 pregnant women who fulfilled the criteria, recruited from phase 1 study. Their blood will be further analysed for the variants of the VDR gene [(BsmI(rs1544410), FokI(rs2228570), TaqI(rs731236)] to look for the association

This study expected to provide more evidence for early personalised intervention of vitamin D supplementation due to anticipated individual genetic variability. This antenatal care programme will reduce the government expenditures, reduce maternal and fetal morbidity and mortality while strengthening Malaysia's healthcare system.

C(xii). Detail Planning

(a) Research background

1. Problem Statement

Gestational hypertension (GH), preeclampsia (PE) and eclampsia are among the major complications that account for approximately 14% of maternal mortality globally (WHO 2016). Preeclampsia complicates 10% of all pregnancies worldwide with 16% maternal deaths globally and 9% mortality rates in Asia and Africa (WHO, 2016). In Malaysia, the frequency of this serious complication is estimated at an incidence rate of 3-4% (National Obstetrics Register Malaysia). GH and PE are also associated with an increased risk of several chronic disorders in mothers later in life, such as cardiovascular diseases, chronic arterial hypertension, stroke, metabolic syndrome, and chronic kidney disease. Infants born to women with PE are at increased risk of cardiovascular diseases and hypertension, insulin resistance, diabetes, and even neurodevelopmental disorders (Bokslag et al., 2016; Mc Kinney et al., 2016; Fox et al., 2019), thereby diminishing productivity of the country's workforce.

Vitamin D deficiency has been recognised as a global health problem, affecting people across all ethnicities and age groups in the world (Nair and Maseeh, 2012). Vitamin D deficiency is defined as plasma/serum levels of 25(OH) D below 20 ng/mL (50 nmol/L) (Hosein-nezhad and Holick, 2013). A low serum level of 25(OH)D has been described as completely preventable (Kiely, 2016). It is regarded as an important public health priority, considering the adverse health outcomes associated with its deficiency state (Munns et al., 2016). Vitamin D has been known to play a crucial role in both genomic and non-genomic function such as bone development and modulation of neurogenesis in developing brain respectively (Xiaoying Cui et al; 2017). This plays a significant role in maternal and child health. Vitamin D deficiency in pregnant mother results in negative health consequences including GH and poor health outcomes to the child with potential to develop life-long chronic diseases in the future. It has been reported that, the prevalence of vitamin D deficiency ranges from 51.3% to 100% among Asians, Middle-East and African countries (van der Plight et al., 2018).

More evidence suggests that genetic variability involving gene polymorphisms and mutations of specific maternal susceptibility genes such as the vitamin D receptor (VDR) gene plays a vital role in the pathogenesis of various complications of pregnancy including hypertensive disorders (Caccamo et al., 2020). Certain VDR gene, BsmI in particular can significantly reduce the effectiveness of vitamin D action, leading to vitamin D deficiency resulting in the development GH and PE (Caccamo et al., 2020). Pregnant women with polymorphic VDR gene variants with low physical activity and sunlight exposure suffer more consequences associated with vitamin D deficiency. This includes impaired calcium and phosphorus ion absorption compared to pregnant women with normal VDR gene. This category of pregnant women need to be identified earlier during the pregnancy so that urgent intervention measures such as personalised form of vitamin D supplementation strategy can be instituted, health education and awareness on sunlight exposure and physical activity will be encouraged. Hence, it plays an important factor of individual susceptibility to biologic effect of vitamin D.

To our knowledge, there has been no published study conducted in Malaysian population on the association of VDR genetic variation and hypertensive disorders of pregnancy among pregnant women in Malaysia, the influence of genetic variability and the role of BsmI, ApaI, TaqI and FokI polymorphisms in the development of these disorders. Therefore, to address these gaps, we aim to investigate the role of VDR genetic variation in the development of GH among Malaysian pregnant mothers, representing the largest ethnicity (69%) in Malaysia (R.Hirschmann, 2021).

2. Hypothesis

1.There is high prevalence of vitamin D deficiency among Malaysian pregnant women.

2.There are genetic variations in the VDR gene fragments (BsmI, ApaI, TaqI and FokI) among Malay pregnant mothers having vitamin D deficiency and developing gestational hypertension.

3. Research Questions

1. What is the prevalence of vitamin D deficiency and associated risk factors among Malaysian pregnant mothers?

2. Is there mutation in the VDR gene (BsmI, ApaI, TaqI and FokI) fragments of vitamin D deficient Malay pregnant women with gestational hypertension?

3. What is the association of vitamin D deficiency and VDR gene polymorphisms in Malay pregnant women with gestational hypertension?

4. Literature Reviews

Gestational hypertension (GH) is defined as an increase in blood pressure greater than 140/90 mmHg measured on two consecutive occasions, between 4-6 hours apart and occurring after 20 weeks of pregnancy. It often presents with complications such as preeclampsia (PE), eclampsia or HELLP syndrome (Caccamo et al., 2020). More evidence suggests that genetic variability such as polymorphisms and mutations of specific maternal susceptibility genes such as the VDR gene plays a pivotal role in the pathogenesis of various disorders including gestational pregnancy (Lee et al., 2020).

Vitamin D is a pre-pro-hormone synthesized from 7-dehydrocholesterol of the epidermis of the skin after exposure to ultraviolet (UV) light from the sun or obtained from the diet. Vitamin D is transported to the liver for hydroxylation to 25(OH) D, the main circulating form of vitamin D (a marker for vitamin D status) and then to the kidney where the biologically active hormonal form, 1, 25(OH)2 D is produced [4]. It is a pleiotropic pro-hormone that functions through an endocrine (regulation of calcium absorption) and autocrine (facilitation of gene expression) mechanism (Jones et al., 1998; Verstuyf et al., 2010).Vitamin D deficiency has been defined as plasma/serum levels of 25(OH) D below 20 ng/mL (50 nmol/L) (Hosein-nezhad and Holick, 2013). Upon ingestion or synthesis by the body through exposure to sunlight, vitamin D is transported to the liver for hydroxylation to 25(OH)D, the main circulating form of vitamin D (marker for vitamin D status) and then to the kidney where the biologically active hormonal form, 1,25(OH)2D is produced.

In pregnant mothers, maternal 25(OH)D can freely cross the human placenta where the placenta expresses vitamin D receptors (VDR) and the enzyme CYP27B1 that can convert 25(OH)D to its biologically active form 1,25-dihydroxycholecalciferol (1,25(OH)2D) (van der Plight et al., 2018).

VDR is a protein comprising of two functional domains (N-terminal dual zinc finger DNA binding domain and C-terminal ligand-binding activity domain) and linking region (Margolis and Christakos, 2010; Pike and Meyer, 2010). VDRs are widely expressed in different tissues. The gene encoding VDR is located on chromosome 12 (12q12-14) (Szpirer et al., 1991; Karonova et al., 2018). Several single nucleotide polymorphisms (SNPs) in the VDR gene associated with metabolic disorders and vitamin D deficiency have been described (Uitterlinden et al., 2004). SNPs, including rs1544410 (BsmI), rs7975232 (ApaI), and rs731236 (TaqI), located at the 3′ untranslated region of VDR gene have been shown to influence mRNA stability and VDR expression (Ogunkolade et al., 2002; Uitterlinden et al., 2004), whereas rs2228570 (FokI) SNP located near the promoter region results in altered VDR activity due to change in amino acid sequence of this protein (Whitfield et al., 2001).

Genetic variants in VDR gene associated with dysregulation of metabolic biomarkers such as anthropometric parameters related to obesity, insulin resistance, Type 2 diabetes mellitus, and atherogenic lipid abnormalities in different populations have been reported (Schuch et al., 2013; Karonova et al., 2018). This could possibly translate to complications in maternal pregnancy with adverse health consequences on both mother and foetus. Vitamin D exerts its effect through the nuclear VDR common SNPs (BsmI, ApaI, and TaqI) found on the 3′ untranslated region (UTR) of the VDR gene. It has been shown to influence mRNA stability and VDR expression, whereas FokI SNP located near the promoter region results in altered VDR activity due to change in the amino acid sequence of the protein (Megan et al., 2009). FokI polymorphism of the VDR gene is also associated with upregulation of angiotensin II type I receptor and renin gene transcription leading to hypertension. BsmI mutated allele affects VDR messenger RNA (mRNA) stability leading to a reduction of VDR protein amount in tissues (Rezavand et al., 2020).

BsmI, ApaI, TaqI and FokI VDR variants have been strongly reported to affect vitamin D binding and are associated with the risk of hypertension (Lee et al., 2020). FokI polymorphism of VDR is also associated with upregulation of angiotensin II type I receptor and renin gene transcription leading to hypertension. BsmI mutated allele affects VDR mRNA stability leading to a reduction of VDR protein amount in tissues. It has been shown that an unfavourable VDR genetic background can significantly reduce the effectiveness of vitamin D action thereby contributing to the development of several disorders including GH (Lin et al., 2019).

Although these data suggest association of vitamin D, VDR gene and development of GH, there was lack of emphasis on this in our current healthcare setting. Malaysian pregnant women were not risk stratified, tested or supplemented with vitamin D accordingly. Thus there is a need to further research this area in our population to determine the prevalence and its association. First part of our study will look into the prevalence of vitamin D deficiency among Malaysia pregnant women. Malay representing the largest ethnic in Malaysia (69%) will be the focus of the second part of our study to avoid bias due to different genetic variation in different ethnicity (R.Hirschmann, 2021).

5. Relevance to Government Policy (if any)

MALAYSIA-WORLD HEALTH ORGANISATION (WHO) -MILLENNIUM DEVELOPMENT GOAL 5 (MDG5)
Sustainable Development Goal 3 (SDG3) focused on good health and well-being together with Millennium Development Goals 5 (MDG 5) focused on improvement of maternal health through efforts to: improve women's access to productive resources, improve women's nutritional status; and empower women for a better health care, education and social services.This goal can be achieved by providing strong evidence for policy formulation such as a need for introduction of health education strategy and vitamin D supplementation programme in the existing antenatal care settings in Malaysia (as seen in the UK, Canada, Sweden, Finland and Australia).

MINISTRY OF HEALTH- PREGNANCY HEATLH FOR MOTHER
Addressing the quality of antenatal care by preventing diseases in pregnancy, thus reducing the financial and health burden of the government. This reduces maternal and child morbidity and mortality whilst further strengthen Malaysia’s health systems.

MINISTRY OF EDUCATION
Strengthening the higher education system by uprising the academic community to recruit, develop and retain outstanding talents in making global recognition.

KEY ECONOMIC GROWTH ACTIVITIES (KEGA)
Allowing for advanced and modern services to be utilised in our health care in particular the women's health.

(b) References

Bokslag, A., van Weissenbruch, M., Mol, B. W., de Groot, C. J. M. (2016). Preeclampsia; short and long-term consequences for mot her and neonate. Early Hum. Dev., 102, 47–50.
Caccamo, D., Cannata, A., Ricca, S., Catalano, L. M, Montalto, A. F., Alibrandi, A., et al. (2020). Role of Vitamin-D Receptor (VDR) single nucleotide polymorphisms in gestational hypertension development: A case-control study. PLoS ONE, 15 (11): e0239407.

Fox, R., Kitt, J., Leeson, P., Aye, C. Y. L., Lewandowski, A. J. (2019). Preeclampsia: Risk factors, diagnosis, management and the cardiovascular impact on the offspring. J. Clin. Med., 8, 1625.

Hosein-nezhad and Holick M. F. (2013). Vitamin D deficiency: A Global perspective. Mayo Clinical Proclamaton, 88(7): 720–755. doi: 10.1016/j.mayocp.2013.05.011.

Karonova, T., Grineva, E., Belyaeva, O., Bystrova, A., Jude, E. B., Andreeva, A, Kostareva A and Pludowski P (2018) Relationship Between Vitamin D Status and Vitamin D Receptor Gene Polymorphisms With Markers of Metabolic Syndrome Among Adults. Front.

Endocrinol. 9:448.doi: 10.3389/fendo.2018.00448

Lee, S. S, King-Hwa, L., Maiza, T., Subramaniam, R., Kartini, F. R. and Loh, S. P. (2020). Influence of vitamin D binding protein poly morphism, demographics and lifestyle factors on vitamin D status of healthy Malaysian pregnant women. BMC Pregnancy and Child birth, 20:714.

Lin, L., Zhang, L., Li, C., Gai, Z., Li, Y. (2019). Vitamin D and Vitamin D Receptor: New Insights in the Treatment of Hypertension. C urr Protein Pept Sci. 2019.

Mc Kinney, D., Boyd, H., Langager, A., Oswald, M., Pfister, A., Warshak, C. R. (2016). The impact of fetal growth restriction on late ncy in the setting of expectant management of preeclampsia. Am. J. Obstet. Gynecol., 214, 395.
Megan, L., Mulligan, B. A, Shaili, K. F., Amy, E. R. and Bernal-Mizrachi, C. (2009). American Journal of Obstetrics and Gynaecology , 202(5):429.

Munns, C. F, Shaw, S, Kiely, M, Specker, B. L., Thacher, T. D., Ozono, K, Michigami, T., Tiosano, D., Mughal, M. Z., Mäkitie, O., Ramos-Abad, L., Ward, L., DiMeglio, L. A., Atapattu, N., Cassinelli, H., Braegger, C., Pettifor, J. M., Seth, A., Idris, H. W., Fu, B. V. J., Goldberg, G., Sävendahl, L., Khadgawat, R, Pludowski, P., Maddock, J., Hyppönen, E., Oduwole, A., Frew, E., Aguiar, M., Tulch insky, T., Butler, G. and Högler, W. (2016). Global Consensus Recommendations on Prevention and Management of Nutritional Rick ets, J Clin Endocrinol Metab, 101(2):394 – 415. doi: 10.1210/jc.2015-2175.

Nair, R. and Maseeh, A. (2012). Vitamin D: The "sunshine" vitamin. J Pharmacol Pharmacother, 3:118-26.

Ogunkolade, B.,W., Boucher, B. J., Prahl, J., M., Bustin, S., A., Burrin, J., M., Noonan, K et al. (2002). Vitamin D receptor (VDR) m RNA and VDR protein levels in relation to vitamin D status, insulin secretory capacity, and VDR genotype in Bangladeshi Asians. Dia betes, 51:2294–300. doi: 10.2337/diabetes.51.7.2294.

R.Hirschmann. (2021). Breakdown of population by ethnicity Malaysia 2019-2021. https://www.statista.com/statistics/1017372/malay sia-breakdown-of-population-by-ethnicity/. Assessed on 30th Jan 2022.

Rezavand, N., Tabarok, S., Rahimi, Z., Vaisi-Raygani, A., Mohammadi, E., Rahimi, Z. (2019). The effect of VDR gene polymorphism s and vitamin D level on blood pressure, risk of preeclampsia, gestational age, and body mass index. Journal of Cell Biochemistry, 1 20:6441-6448.

Schuch, N., J., Gastina, V. C., Vivolo, S. R. G. F., Martini, L. A. (2013). Relationship between vitamin D receptor gene polymorphis ms and the components of metabolic syndrome. Nutr J., 12:96. doi: 10.1186/1475-2891-12-96.

Szpirer, J., Szpirer, C., Riviere, M., Levan, G., Marynen, P., Cassiman, J., et al. (1991). The Sp1 transcription factor gene (SP1) and the 1,25-dihydroxyvitamin D3 receptor gene (VDR) are colocalized on human chromosome arm 12q and rat chromosome 7. Genomic s, 11:168–73.

Uitterlinden, A. G., Fang, Y., van Meurs, J. B. J, Pols, H. A. P, van Leeuwen, J. P. T.M. (2004). Genetics and biology of vitamin D r eceptor polymorphisms. Gene, 338:143–56. doi: 10.1016/j.gene.2004.05.014.

Van der plight, P., Willcox, J., Szymlek-Gay, E. A., Murray, E., Worsley, A. and Daly R. M. (2018). Associations of Maternal Vitamin D Deficiency with Pregnancy and Neonatal Complications in Developing Countries: A Systematic Review. Nutrients, 10, 640; doi:10. 3390/nu10050640.

Whitfield, G., K., Remus, L., S., Jurutka, P., W., Zitzer, H., Oza, A., K., Dang, H. T., et al. (2001). Functionally relevant polymorphis ms in the human nuclear vitamin D receptor gene. Mol Cell Endocrinol. 177:145–59. doi: 10.1016/S0303-7207(01)00406-3.

World Health Organisation (WHO). Maternal Mortality Fact sheets Report. 19th September, 2019.
World Health Organization. Fact Sheet - Maternal Mortality. http://www.who.int/mediacentre/factsheets/fs348/en/. Accessed 23 July 2021.

(c) Objective(s) of the Research

General objective:

To investigate the prevalence of vitamin D deficiency and the association of VDR SNPs to the development of GH among Malay pregnant mothers

Specific objectives:

1.To determine the prevalence of vitamin D deficiency and associated risk factors among Malaysian pregnant mothers through a cross-sectional study.
2. To understand and associate distributions of VDR allele and genotype with vitamin D deficiency.
3. To associate VDR-combined genotypes with the risk to develop GH in vitamin D deficient Malay pregnant women.

(d) Methodology:

1. Description of Methodology

Study Duration
The study will be conducted within 36 months (3 years), between Sept 2022 to August 2025.

Study location
The study will be carried out in Hospital Pengajar UPM (HPUPM), Hospital Serdang in Selangor, Malaysia and Medical Genetics Lab in the Faculty of Medicine & Health Sciences of Universiti Putra Malaysia.

Study Population
All Malaysia pregnant women attending Obstetrics and Gynaecology Department of Hospital Pengajar UPM(HPUPM) and Hospital Serdang who fulfilled the criteria will be recruited.

Study Design
This is a prospective study and it will be divided into two phases

1) Cross-sectional study (Phase 1)

Objective 1: To determine the prevalence of vitamin D deficiency and the associated risk factors among Malaysian pregnant mothers through a cross-sectional study.

Aim: This part of study will gather information on clinical, socio-demographic, dietary intake and anthropometric data (height, weight and BMI) using a validated questionnaire to determine the risk factors associated with vitamin D deficiency. Vitamin D status (25-hydroxycholecalciferol (25(OH)2 D) will be determined by measuring the vitamin D level in the serum by withdrawing about 10mls of blood and for vitamin D status, will be analyzed using the gold standard method of liquid chromatography-tandem mass spectrometry (LC-MS/MS).

Sampling method:
A simple random sampling method will be used to recruit the study participants for the cross-sectional study.

Inclusion criteria
1. Malay, Chinese and Indians race
2. Pregnant women with gestational age between 20 weeks till 42 weeks. (The gestational age will be determined from the first day of the last menstrual cycle or measurement of fetal crown rump length)
3. Pregnant women with viable pregnancy at the time of recruitment.
4. Literate in either English or Malay languages.
5. Agreement to participate and sign the written informed consent.

Exclusion criteria
1. Women already on vitamin D supplements or any other drugs that can interfere with vitamin D absorption or metabolism at the time of recruitment.
2. Pregnant women with any other chronic disease that is known to affect vitamin D such as, autoimmune disease, cancer, etc.

Other variables:

Body Mass Index Calculation (BMI)
The pre-pregnancy BMI will be calculated from pre-pregnancy body weight recall or obtained from the woman's antenatal booking record and the measured height. The pregnancy BMI will be measured using the pregnancy weight and the measured height. The BMI (kg/m2) will be calculated by dividing the weight (kg) by the square of the height (m2).
BMI (kg/m2) = Weight (kg) / Height (m2).

Expected results and statistical analysis:
The prevalence of vitamin D deficiency will be obtained by recording the frequency and percentage. Other variables including dietary intake, anthropometric data will be collected and tested for normality and the association with socio-demographic data will be analysed using the chi-square test, independent t-test and ANOVA.

2) Case-control study (Phase 2)

Objective 2: To understand and associate distributions of VDR allele and genotype with vitamin D deficiency among Malay pregnant mothers

Aim: This part of the experiment aims to determine the VDR genetic variants and the genotypes among vitamin D deficient Malay pregnant women with (case) and without (control) hypertension using high-resolution melting (HRM) analysis in real-time polymerase chain reaction (RT-PCR). The four commonly reported VDR polymorphisms (BsmI, ApaI, TaqI and FokI) will be determined using the PCR-HRM platform using Light-Cycler 480 (Roche) from genomic DNA extracted from patients' blood. This case-control study will involve 90 vitamin D deficient Malay pregnant women with GH and 90 without GH, recruited from phase 1.

Sampling Method
Purposive sampling method will be used to recruit subjects for the case-control study.

Inclusion criteria
1. Malay pregnant women with gestational age between 20 weeks till 42 weeks. (The gestational age will be determined from the first day of the last menstrual cycle or measurement of fetal crown rump length)
2. Malay pregnant women with viable pregnancy with or without pregnancy induced hypertension or its complications (pre-eclampsia,

eclampsia, HELLP syndrome etc).
3. Literate in either English or Malay languages.
4. Agreement to follow-up till delivery and provision of written informed consent.

Exclusion criteria
1. Non-viable pregnancy at the time of recruitment.
2. Women already on vitamin D supplements or any other drugs that can interfere with vitamin D absorption or metabolism at the time of recruitment.
3. Women with chronic hypertension diagnosed prior to pregnancy.
4. Pregnant women with any other chronic disease that is known to affect vitamin D such as, autoimmune disease and any malignancy

Justification to use PCR-HRM analysis:
HRM is a simpler and more cost-effective analysis for characterizing samples than probe-based genotyping assays. It provides a closed and direct assay system that does not require post-PCR processing. HRM results are comparable to more time-consuming and expensive conventional methods such as single-strand conformation polymorphism, denaturing high-pressure liquid chromatography, restriction fragment length polymorphism, and DNA sequencing.

Genomic DNA extraction
Genomic DNA will be extracted from the peripheral blood monocytes using Picoll's floatation method and stored at -20˚C until analysis. The polymerase chain reaction (PCR) technique will be used to amplify the VDR gene by using specific primers. Genomic DNA will be extracted from the patient's blood using DNeasy Blood & Tissue Kits (Qiagen) following the manufacturer's instructions. The extracted DNA will be quantifies using Nanovue (GE Healthcare) and quantitatively assessed on agarose gel before being subjected to PCR-HRM analysis.

Primer design:
Four sets of primers will be synthesized based on published sequences (Divanoglou et al., 2021, Table 1). These primers will be used to run PCR-HRM analysis to detect the polymorphisms of the VDR variants.

Refer Table 1

PCR-HRM Cycling conditions:
The extracted DNA will be subjected to PCR-HRM following the manufacturer's protocol using the cycling conditions based on previously published paper (Divanoglou et al., 2021, Table 2).

Refer Table 2

BT-Seq Analysis:
Next-Generation-Sequencing (NGS)-based Barcode-Tagged sequencing (BTSeq, Celemics) analysis will be used to verify the single nucleotide polymorphisms (SNPs) of the VDR gene variant(s) among vitamin D deficient Malay pregnant mothers with GH obtained from the PCR-HRM results. BT-Seq provides rapid and more accurate sequences compared to the conventional Sanger method. This part of the experiment will involve 10 samples out of 180 samples as representative samples to act as the reference genotype for all SNPs detected by the PCR-HRM analysis.
The analysis will be done on each VDR variant amplicon that will be synthesized based on published primers spanning the four VDR SNPs (Divanoglou et al., 2021, Table 3) using PCR. The amplicons will be run on agarose gel and cut purified from the gel before sending for sequencing (BT-Seq).

Refer Table 3

Expected results and analysis:
Discrimination of the three possible genotypes of each polymorphism (common homozygotes, heterozygotes, and rare homozygotes) in 3 distinct groups will be obtained from 180 samples by PCR-HRM analysis. Validation of the polymorphisms obtained from the PCR-HRM analysis will be done on 10 representative samples by NGS-based BT-Seq analysis. A T→C transition in introns 8 and 9 will reflect the presence of the SNPs in intron 8 and 9 for BsmI and TaqI, respectively. A C→T transition at the junction of intron 1 and exon 2 reflects the FokI variant, and the ApaI variant is reflected by a T→G transition in intron 8.
Distribution of vitamin D status and the genotype frequency with Hardy-Weinberg equilibrium will be obtained for each SNP. The significant p-value be calculated using one-way ANOVA. The correlations of VDR polymorphisms with the risk of low serum vitamin D levels will be determined using logistic regression analysis.

Objective 3: To associate VDR-combined genotypes with the risk to develop GH in vitamin D deficient Malay pregnant women

Aim: To determine the association between vitamin D status, the haplotypes subgroups and the genotype of BsmI, ApaI, TaqI and FokI VDR gene variants among vitamin D deficient Malay pregnant women with and without hypertension through a case-control study.

Expected result and statistical analysis:
Correlation and regression model will be applied to evaluate a possible significant dependence of GH from the 25(OH)vitamin D3 levels from haplotypes subgroups.

Ethical Consideration
Permission to carry out the study will be obtained from the ethics and research committee involving human subjects of the Universiti Putra Malaysia (JKEUPM) and the Medical Research and Ethics Committee (MREC). The study will be conducted in accordance

with the standards of human experimentation in the Helsinki Declaration of 1975, as revised in 2000. Informed consent will be sought from all eligible participants.

Sample Size and Population
A total of 363 pregnant women will be recruited for the Cross-sectional study (Phase 1) and will be used to determine the prevalence of vitamin D deficiency and associated risk factors (objective 1).
For Phase 2, which is the case control study, total of 90 Malaysian pregnant women with gestational hypertension (case) and a total of 90 non-hypertensive vitamin D deficient pregnant women will be recruited as a control.

Sample size Determination

Sample size Determination for Cross-sectional study (Phase 1- objective 1)
The sample size for the study will be calculated using the following formulae (Cochran, 1977).
n = Z2 pq
d2
Where:
n = Minimum number of sample size
Z = Level of significance at 95% confidence interval (1.96)
p = Prevalence rate
q = 1- p
d = Tolerable margin of error (5%) = 0.05
According to Woon et al., (2019), the prevalence of vitamin D deficiency among Malaysian pregnant women is 42.6%.

n = Z2 pq
d2
= (1.96)2 × 0.426 (1-0.426)
(0.05)2

= 3.8416 × 0.426 × 0.574
0.0025

= 329.85

Therefore, the minimum sample size required for the study after addition of 10% attrition rate is approximately is 363.

Sample size Determination for Case-Control study (Phase 2-objective 2-5)
The minimum number of subjects that will participate in the study will be determined by the formula below according to Charan and Biswas (2013).
n = r+ 1 P* (1- P*) (Zβ + Zα/2) 2
r (P1 – P2) 2
According to a study by Caccamo et al., (2020), the prevalence of vitamin D among women with gestational hypertension and pregnant women without hypertension is 21% and 11% respectively.
Zβ = Power (80%) = 0.84
Zα/2 = For 0.05 significance level, for 95%= 1.96
P1 = Proportion exposed in the cases=21%= 0.21
P2 = Proportion exposed in the controls=11%= 0.11
Effect size (P*) = P1 - P2
= 0.21- 0.11
= 0.1
n = (1+1) (0.1) (1- 0.1) (0.84+1.96)2
1 (0.1)2

= 81.846
If we add 10% attrition to the calculated Sample size, our minimum number of subjects in the cases will be 90.
Since the ratio of control to case is 1, the minimum number of controls will be 90 also.

2. Flow Chart of Research Activities	'Flowchart final.pdf'

3. Research Activities

Activity	Start Date	End Date
Ethical approval	01/09/2022	30/11/2022
Participants recruitment and sample collection	01/12/2022	30/11/2023
Vitamin D status quantification	01/01/2023	31/12/2023
DNA extraction and quantification	01/04/2023	31/03/2024
Real time PCR and HRM analysis	01/05/2023	30/04/2024
BT-Seq	01/07/2023	30/06/2024

Association and correlation studies	01/07/2024	28/02/2025
Manuscript writing, presentation at conference and publication	01/01/2025	31/08/2025

4. Milestones

Description	Date	Cumulative Project Completion Percentage(%)
Obtained ethical approval	30/11/2022	5
Completion of sample collection for phase 1	30/11/2023	30
Completion of sample collection for phase 2	31/03/2024	60
Completion of PCR-HRM analysis to determine VDR variants	30/04/2024	70
Completion of BT-Seq to determine the genotype	28/02/2025	80
Completion of the association and correlation analysis	30/04/2025	90
Completion of manuscript writing	31/08/2025	100

Gantt Chart of Research Activities with Milestones

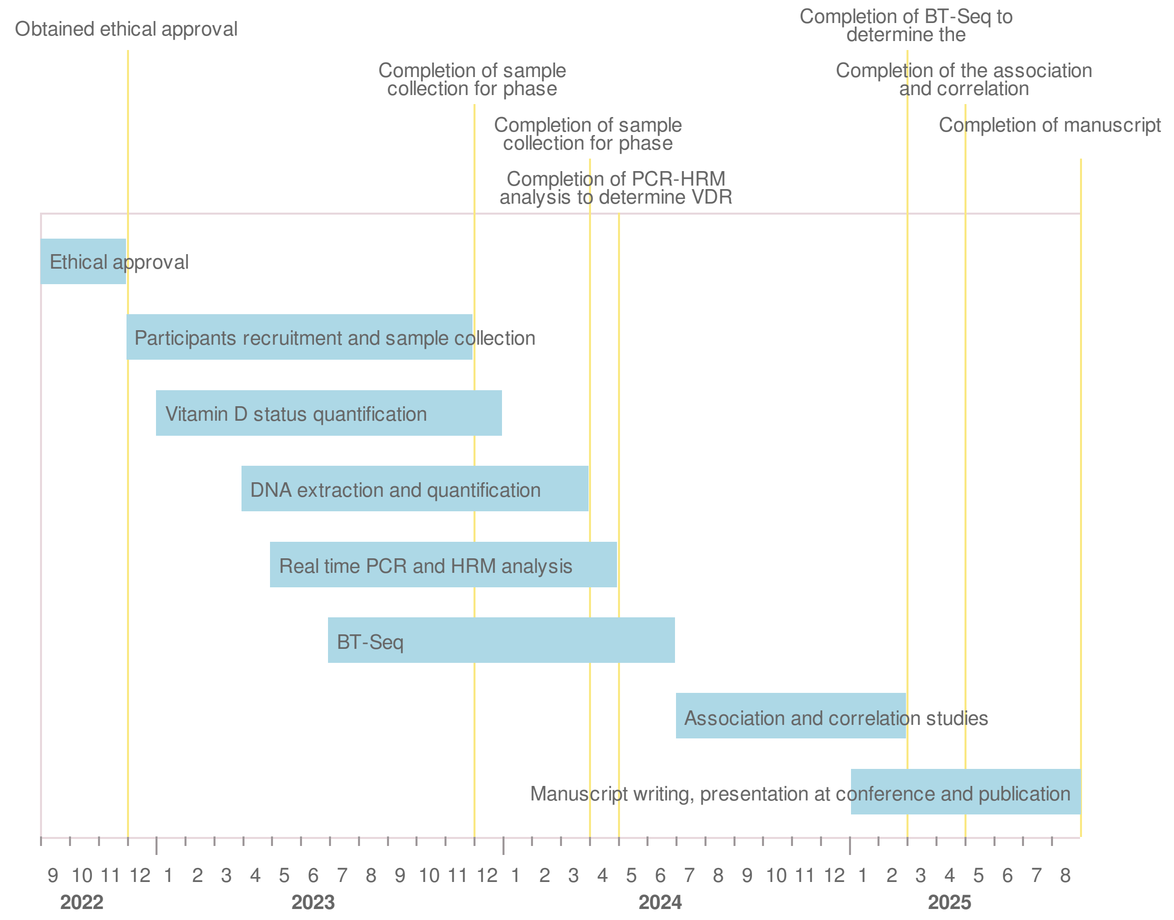

(e) Expected Results/Benefit

1. Novel theories/New findings/Knowledge

1. The prospective project will provide excellent opportunities to link molecular understanding of the role of VDR genetic variation and could be a risk factor for the development of GH

2. The findings of the study could reveal the possible association of VDR gene variants with higher risk GH development among Malay pregnant women in Malaysia.

3. The study will deepen our understanding of variability in the encoding genes of vitamin D. This could further find application in risk assessment of GH and predicting response to treatment.

4. The result may contribute to the body of knowledge in women & child healthcare.

2. Impact Statement on Quintuple Helix (please delineate/describe expected research deliverables on Society, Academia, Government, Industry and Environment)

1. The outcome will contribute to the understanding of the role of genetic variability among academia, provide more evidence of a need for customized antenatal care which is valuable to the society.

2. To provide evidence for policy formulation by the introduction of health education strategy and vitamin D supplementation programme in the existing antenatal care in Malaysia.

3. Utilisation by manufacturers of sugar, milk and flour, with a view to evaluate the vitamin D composition of these food products and work towards increasing vitamin D fortification of these products.(industry)

4. It may open up foster collaboration of stakeholders from the academia, government and non-governmental organizations, industries and civil society groups with a view to establish “The Vitamin D Council” in Malaysia (as obtainable in UK, Canada, Sweden, Finland and Australia), with a goal to educate general public, promote and coordinate research activities linking vitamin D with human diseases.

3. Research Publications (Each proposal must produce at least two (2) papers in indexed journals, one of which should be in Web of Science (WoS))

**Indexing Body**	**Indexed Journal**	
**Number of Publication**	**Name of Journal**	
WoS	2	1. BJOG-International Journal of Obstetrics and Gynaecology<br>Quartile: Q1<br>Impact Factor: 6.531<br><br>2.BMC Pregnancy and Childbirth, Quartile Q2<br>Impact Factor 3.86
SCOPUS		
ERA		
MyCITE		
	**Total 2**	

4. Specific or Potential Applications of the Research Findings

1. To contribute for the establishment of a sustainable regional and national intervention scheme for testing/screening, prevention (through fortification of foods such as milk, flour, sugar, infant feeds etc.) and treatment through supplementation of vitamin D with a tailored or customised form in anticipated individuals with genetic variability with potential use for women and child health well being.

2. Introduction of vitamin D supplementation programme in the existing antenatal care settings. To bring together relevant stakeholders in the food and drug administration agencies, food manufacturing industries (manufacturers of milk, flour, sugar and infant feeds), development partners and government health authorities with a view to further evaluate vitamin D composition of these products and work towards increasing vitamin D fortification of these foods.

Total Number of Applications: 2

5. Number of PhD and Masters (by research) Students

Total Number of PhD (by research) Student(s):
1

Total Number of Masters (by research) Student(s):
0

Remark (if any):

6. Intellectual Properties (IPs)

Total Number of IP: 0

Access to Equipment & Material(s)

**Type**	**Description**	**Owner**	**Location**	**Address**
LCMS/ HPLC Machine	For the analysis of vitamin D, 25(OH)D	Agro-Biotechnology Institute	Kompleks Agro-Biotechnology Institute Malaysia	Jalan Eksotika, 43400, Serdang, Selangor
Gel Electrophoresis Machine	For separation of digested bands	Medical Genetics Lab	Faculty of Medicine & Health Sciences	Universiti Putra Malaysia
-20˚C to -80˚C Liquid [illegible]	For storing of DNA samples [illegible]	Medical [illegible]	Faculty of Medicine & Health [illegible]	Universiti Putra Malaysia

Nitrogen Freezer	For storing of DNA samples	Genetics Lab	Sciences	Universiti Putra Malaysia
LC480 PCR Machine	For gene quantification and detection	Medical Genetics Lab	Faculty of Medicine & Health Sciences	Universiti Putra Malaysia
Nano drop spectrometer	For measuring the purity if extracted DNA	Medical Genetics Lab	Faculty of Medicine & Health Sciences	Universiti Putra Malaysia
4-6˚C Refrigerator	For storing of primers and other consumables	Medical Genetics Lab	Faculty of Medicine & Health Sciences	Universiti Putra Malaysia

F(i). Patent Search (describe how your research output shall produce an innovative idea or technology that has the potential to be a solution for stakeholders (community, industry, government etc.) and offers a unique proposition)

To identify if the researcher is able to coherently present a compelling argument for his/her proposal in light of the IP landscape and factors identified in the (Yes/No) Section. The answer would reflect an understanding of the applicant's research advantage and limitations and the prospect of moving the completed research beyond this stage of funding.

The outcome of the prospective project shall be the first of its kind to detect novel sequences of genetic mutation on VDR gene fragments among Malay population of pregnant mothers with gestational hypertension. Currently there is no patents found regarding the VDR gene among pregnant mothers, not to mention those with gestational hypertension or among Malay population.

These VDR gene novel sequences could be patented, presented to relevant Government authorities and will be utilised in the synthesis of specific primers by diagnostics companies/industries. This will go a long way in promoting research and aid diagnosis of genetic variation or polymorphisms of chronic diseases involving Malaysian population and perhaps, could be utilised by other population.

Moreover, this innovation will enable clinicians to better understand the influence of the novel sequences causing the genetic variation in the development hypertension in pregnancy so that personalized vitamin D supplementation strategy could be recommended for the prevention of gestational hypertension which is a serious challenge in Malaysia. Hence, the future direction of this project is to conduct a randomised controlled clinical trial of vitamin D supplementation of pregnant women to evaluate the effect of individualised treatment and monitor response to the treatment.

Simplified Patent Search Report (MyGRANTS) 2022.pdf

F(ii). Research Collaborator

- ☐ Industrial Linkages (Please identify any industry or end-user group involved in the project, and describe its role/contribution to the project)
- ☐ Agency/Organisation (Please identify all agencies/organisations collaborating in the project, and describe their role/contribution to the project)

F(iii). Risk Assessment (Please describe factors that may cause delays in, or prevent implementation of, the project as proposed above; estimate also the degree of risk)

Please consider an appropriate approach to working in the current conditions (pandemic, travel ban, social distancing etc.)

1. Pandemic and social distancing- participants recruitment will be done face to face during the patients attendance at study location, together with blood collection. Due to the current restriction, questionnaire will be distributed via google forms to reduce the duration of interaction and risk of contracting Covid-19.

Risk	Low	Medium	High
1. Technical	☐	☑	☐
2. Timing	☐	☑	☐
3. Budget	☐	☑	☐

Appendix

Appendix	Name	File Name
A	CV of Principal Investigator	CURRICULUM VITAE 1.2.22.pdf
B	CV of co-investigator	CV Amilia Afzan UPM.pdf
C	CV of co-investigator	CV Norshariza Nordin Jan 2021.pdf
D	CV of co-investigator	Aida Adha.pdf
E	CV of co-investigator	loh su peng.pdf

Application Form Submission

Status	Approved
Application Date	02/03/2022

Overall Remark

Tajuk projek dipinda seperti mana cadangan panel.

This research proposal is sound. Please improve the proposal as stated in the comments below:

The panel noted that there are many published study conducted among the global population population on the association of VDR genetic variation and GH. However we also support the intention of the team of researcher to investigate the prevalence of vitamin D deficiency and its association of VDR SNPs to the development of GH among pregnant mothers in our own country.

We would suggest that the study should cover all races. Later on, the data for each races can be further analysed with regard to the response of GH management in the public, university and private hospitals, especially among the B 40 pregnant mothers.
